# Bushenhuoluo Decoction improves polycystic ovary syndrome by regulating exosomal miR-30a-5p/ SOCS3/mTOR/NLRP3 signaling-mediated autophagy and pyroptosis

**DOI:** 10.1186/s13048-024-01355-x

**Published:** 2024-02-01

**Authors:** Qun Huang, Yuanbin Li, Zhuang Chen, Huiping Ou, Yanjiao Tan, Hui Lin

**Affiliations:** 1 Department of Gynecology, The First Affiliated Hospital of Hunan Traditional Chinese Medical College, Zhuzhou, 412012 Hunan Province People’s Republic of China; 2 Department of Traditional Chinese Medicine, Hunan Traditional Chinese Medical College, No. 88 Zhihui Road, Shifeng District, Zhuzhou, 412012 Hunan Province People’s Republic of China

**Keywords:** PCOS, BSHLD, Exosome, miR-30a-5p, SOCS3, Autophagy, Pyroptosis

## Abstract

**Background:**

Polycystic ovary syndrome (PCOS) is a frequent and complicated endocrine disease that remains a major reason for infertility. Bushenhuoluo Decotion (BSHLD) has been validated to exhibit curative effects on PCOS. This study was aimed to explore the potential mechanism underlying the therapeutic action of BSHLD.

**Methods:**

PCOS rat model was induced by dehydroepiandrosterone (DHEA). Serum hormone and cytokines levels and ovarian pathological alterations were measured to assess ovarian function. Exosomes (Exos) were identified by Transmission electron microscopy and Nanoparticle Tracking Analysis. RT-qPCR, Western blotting, immunohistochemical staining, and immunofluorescence staining were performed to detect molecule expressions. Proliferation and pyroptosis of granulosa cells (GCs) were evaluated by CCK-8 and flow cytometry, respectively. The binding relationship between miR-30a-5p and suppressor of cytokine signaling 3 (SOCS3) was verified by dual luciferase reporter and RIP assays.

**Results:**

BSHLD treatment improved serum hormone abnormality, insulin sensitivity, and ovarian morphologic changes of PCOS rats. Moreover, BSHLD treatment restrained the excessive autophagy and pyroptosis in ovarian tissues of PCOS rats. Moreover, BSHLD reduced the expression of miR-30a-5p in serum, serum-derived Exos, and ovarian tissues, thus inhibiting autophagy and NLRP3-mediated pyroptosis in GCs. Mechanistically, SOCS3 was proved as a target of miR-30a-5p and could activate mTOR/P70S6K pathway to repress autophagy. The inhibitory effect of miR-30a-5p deficiency on autophagy and pyroptosis of GCs was attenuated by rapamycin.

**Conclusion:**

Collectively, BSHLD suppressed autophagy and pyroptosis to improve POCS by regulating exosomal miR-30a-5p/SOCS3/mTOR signaling.

**Supplementary Information:**

The online version contains supplementary material available at 10.1186/s13048-024-01355-x.

## Highlights

1. BSHLD improved the ovarian function and repressed excessive autophagy and pyroptosis in PCOS rats.

2. BSHLD reduced miR-30a-5p expression in serum, serum exosomes and ovarian tissues of PCOS rats.

3. BSHLD relieved the autophagy and pyroptosis by decreasing exosomal miR-30a-5p.

4. miR-30a-5p inactivated mTOR/p70S6K signaling by targeting SOCS3, thus activating autophagy and pyroptosis.

**Supplementary Information:**

The online version contains supplementary material available at 10.1186/s13048-024-01355-x.

## Introduction

Polycystic ovary syndrome (PCOS) is a reproductive-metabolic disorder complex involving multiple systems which mainly occurs in women of reproductive age. PCOS is depicted by sporadic menstruation, body fat and hairy, acanth nigricans, facial acne, infertility, and progressive enlargement of bilateral ovaries [1]. It is a risk factor for insulin resistance (IR), type 2 diabetes, obesity, and cardiovascular disease [2, 3]. For decades, the pathogenesis of PCOS is closely associated to both heredity and environment, but the exact etiology and mechanism remain unclear. Letrozole, clomiphene, and antiandrogens have been clinically used for PCOS patients, however, clinical resistance, multifetation, as well as ovarian hyperstimulation syndrome commonly occur after long-term use [4]. Therefore, it is essential to identify more effective ovulation induction therapy for PCOS patients.

Traditional Chinese medicine (TCM) has been applied extensively in the treatment of PCOS through system assessment of signs and symptoms. For example, Bushen Huatan Decoction has been proved to adjust menstrual cycle, improve insulin resistance and glucose metabolism disturbance in PCOS patients [5]. Bushen Jieyu Tiaochong Formula, another TCM prescription, was reported to recover ovulation in PCOS rats via modulating PERK-ATF4-CHOP pathway [6]. Bushen huoxue formula could improve dehydroepiandrosterone (DHEA)-induced inflammation and endocrine disruption by suppressing TLR4/ NF-κB signaling [7]. Bushen huoluo Decotion (BSHLD) is a traditional empirical formula by which consists of 14 traditional Chinese herbs, including Shudihuang, Danggui, Chuanxiong, Baishao, Nvzhenzi, Hanliancao, Tusizi, Chongweizi, Fupenzi, Yinyanghuo, Taoren, Honghua, Lulutong, and Huainiuxi. Notably, the sovereign drug of BSHLD is Shudihuang, which is considered to nourish Yin and invigorate the kidney in Traditional Chinese Medicine and has widely been used for treating osteoporosis, and cardiovascular disease in China [8]. Danggui and Tusizi are the minister drugs that were used to reinforce kidney essence, promote blood circulation, and strengthen the role of Shudihuang. The compatibility of other drugs can further enhance the therapeutic action of BSHLD. In addition, pharmacological experiment further validated that aqueous extract from Danggui exhibited a beneficial effect on PCOS rats via regulation of the PI3K/AKT, PPAR, MAPK, AMPK, and insulin signaling pathways [9]. Some preliminary studies found that BSHLD treatment could reduce weight, improve serum LH and LH/FSH levels, restore normal menstrual cycle and increase ovulation rate in PCOS rats (Attachment materials). But its specific action mechanism remains to be studied.

Autophagy is an important cellular process that controls cell homeostasis and plays a critical role in cell survival and death. Under normal condition, autophagy can maintain metabolic balance, while excessive autophagy activation can lead to metabolic imbalance and apoptosis [10, 11]. Autophagy is mediated by a series of autophagy-related proteins, such as ATG5, ULK1, Beclin1, LC3I/II, which exert pivotal roles in autophagy initiation, autophagolysosomal maturation, autophagosome membrane expansion and completion [12]. Recent discoveries proved a significant role of autophagy in the pathogenesis of PCOS. It has discovered that the expression of LC3B was increased in the ovarian tissues of PCOS patients and rats, while the level of autophagy substrate SQSTM1/p62 was decreased, suggesting that autophagy was significantly activated in POCS pathology [13]. Repressing the excessive autophagy of granulosa cells (GCs) was considered as a key way to alleviate PCOS [14, 15]. As a key modulatory pathway of autophagy, mechanistic target of rapamycin kinase (mTOR)/p70 Ribosomal protein S6 kinase (p70S6K) has been documented to participate in PCOS progression [16]. Previous studies showed that activation of mTOR/p70S6K restrained excessive autophagy of granulosa cells (GCs) to mitigate PCOS [14, 17]. Moreover, mTOR-mediated autophagy was closely correlated to NLRP3 inflammasome formation and pyroptosis [18, 19]. In addition, NLRP3 inflammasome activation was identified to drive ovarian dysfunction and fibrosis in PCOS mice [20]. However, whether BSHLD exerted protection against PCOS via repressing excessive autophagy and pyroptosis remains unclear.

Exosomes are extracellular vesicles (about 30–150 nm in diameter), which deliver multiple macromolecules, such as proteins, mRNAs, and microRNAs (miRNAs) to recipient cells, and thus play crucial roles in cellular communication [21]. As reported by Zhang et al., a series of miRNAs were differentially expressed in serum exosomes of PCOS patients, which may be used for diagnostic biomarkers for PCOS [22]. Yuan et al. showed that exosomal miR-424-5p derived from PCOS follicular fluid could promote GC senescence by repressing CDCA4 [23]. In addition, a previous study documented that circulating miR-30a was up-regulated in follicular fluid exosomes of PCOS patients, and the area under the ROC curve was 0.67, which indicated an excellent diagnostic value [24]. However, the role of miR-30a-5p in PCOS remains largely unknown.

To the best of our knowledge, this investigation is the first to prove the roles and mechanism of BSHLD on the autophagy and pyroptosis of GCs in PCOS. Our findings demonstrated that BSHLD improved ovarian function and restrained excessive autophagy and pyroptosis by modulating the exosomal miR-30a-5p/ suppressor of cytokine signaling 3 (SOCS3)/mTOR/NLRP3 axis. This work would provide some theoretical and data support for the roles of BSHLD in PCOS.

## Methods

### Animal model

The animal experiments were conducted according to the National Institutional Guidelines for the Care and Use of Laboratory Animals and approved by the Ethics Committee of The First Affiliated Hospital of Hunan Traditional Chinese Medical College. Female Sprague–Dawley rats (150–200 g) were provided by Beijing Vital River Laboratory Animal Technology Co., Ltd (Beijing, China) and randomly divided into sham, dehydroepiandrosterone (DHEA), DHEA + BSHLD groups (*n* = 10 per group). PCOS was induced in rats by daily subcutaneous injection with DHEA (6 mg/100 g, Merck, Darmstadt, Germany) dissolved in 0.2 ml sesame oil for 20 consecutive days. The sham rats were subcutaneously injected with 0.2 ml sesame oil. After DHEA injection (day 21), the rats in DHEA + BSHLD group were administrated with BSHLD (18 g/kg, The First Affiliated Hospital, Hunan University of Traditional Chinese Medicine) by gavage once a day for 14 days. The rats in sham and DHEA groups were administrated with distilled water. After BSHLD treatment, the rats were weighed, followed by euthanasia via cervical dislocation. The ovaries and serum samples were collected. The ovary weight/body weight was used to calculate the ovarian index.

### Detection of serum hormones, fasting plasma glucose, and insulin levels

The serum levels of testosterone (ab285350, Abcam, UK), estradiol (E2, ab285285, Abcam), luteinizing hormone (LH, CEA441Ra, USCN Life Science and Technology Company, Wuhan, China), follicle-stimulating hormone (FSH, CEA830Ra, USCN), and fasting insulin (FINS, ab273188, Abcam) were detected using commercial ELISA kits following the manufacturer’s protocols. Fasting plasma glucose (FPG) level was assessed with a blood glucose meter (Omron, USA). Homeostasis model assessment of insulin resistance (HOMA-IR) = FINS × FPG/22.5.

### Hematoxylin–eosin (HE) staining

The ovary tissues were fixed with 4% paraformaldehyde, embedded in paraffin, sectioned into 4-μm slices, and subjected to staining with the Hematoxylin and Eosin Staining Kit (Beyotime, Shanghai, China). The stained slices were examined under a light microscope (Olympus, Tokyo, Japan). As previously reported [25, 26], the number of primordial, primary, secondary, cystic follicles, antral follicles and corpus luteum in the ovary sections was counted. Primordial follicles were defined as containing an oocyte surrounded by a single layer of squamous granulosa cells. Primary follicles were defined as an oocyte surrounded by prismatic single or multi-layered granulosa cells. Secondary follicles were defined as covering by more than one layer granulosa cells with a small antrum. Cystic follicles were defined as containing fragmented granulosa cells and a thin granulosa cell layer. Antral follicles were identified as a follicle with an antral cavity, regardless of their size. The same sections were independently evaluated by two observers who were blinded to the groups.

### Immunohistochemical staining

For immunohistochemical staining, the ovary slices were immersed in citrate buffer for antigen retrieval. Then, sections were incubated with 3% hydrogen peroxide for 10 min under dark condition at room temperature. The primary antibodies against NLRP3 (MA5-32,255, Thermo Fisher, MA, USA) and anti-LC3B (PA1-46,286, Thermo Fisher) was incubated with sections at 4 °C overnight, and the goat anti-rabbit secondary antibody (ab207995, Abcam) was also probed for 60 min. Subsequently, the slices were visualized with diaminobenzidine and observed under a light microscope.

### Detection of IL-1β and IL-18 levels

The levels of IL-1β and IL-18 in rat ovaries were measured using the Rat IL-1 beta ELISA Kit (ab255730, Abcam) and Rat IL-18 ELISA Kit (ab213909, Abcam) following the instructions, respectively. Optical density values were determined with a plate reader.

### Reverse transcription quantitative PCR (RT-qPCR)

Total RNA was isolated using TRIzol reagent (Thermo Fisher) and reverse transcribed into cDNA using the Reverse Transcriptase Kit (M-MLV) (Invitrogen, Carlsbad, CA, USA). Then, the samples were detected with the SYBR Green qPCR Master Mix (Thermo Fisher). Relative gene expression was determined using the 2^−*ΔΔ*Ct^ method and normalized to GAPDH or U6. The primer sequences were showed in Table 1.

**Table 1:** Primer sequences for RT-qPCR

Gene	Forward (5’-3’)	Reverse (5’-3’)
NLRP3	GTAGGTGTGGAAGCAGGACT	CTTGCTGACTGAGGACCTGA
ASC	ACAGTACCAGGCAGTTCGTG	GGTCTGTCACCAAGTAGGGC
Caspase 1	TCCTGAGGGCAAAGAGGAAG	CACAGGTCTCGTGCCTTTTC
SOCS3	TACTGAGCCGACCTCTCTCC	TGGGGCTGGATTTTTGTGCT
miR-30a-5p	GGGCCTGTAAACATCCTCG	GAATACCTCGGACCCTGC
U6	GTGCAGGGTCCGAGGT	CTCGCTTCGGCAGCACA
GAPDH	GCAAGTTCAACGGCACAG	GCCAGTAGACTCCACGACAT

### Western blotting

Total protein samples were obtained after lysing in RIPA lysis buffer (Yeasen, Shanghai, China). The concentration was examined by a BCA kit (Beyotime Biotechnology, Shanghai, China). The quantified protein was subjected to sodium dodecyl sulfate polyacrylamide gel electrophoresis and transferred to polyvinylidene fluoride membranes (PVDF, Millipore, Boston, MA, USA). After blocking in 5% skimmed milk, the membranes were probed with primary antibodies LC3II/I (bs-8878R, Bioss, Woburn, MA, USA), Beclin1 (bs-1353R, Bioss), ATG5 (bs-4005R, Bioss), ULK1 (bs-3602R, Bioss), p-mTOR (bs-3495R, Bioss), mTOR (bs-1992R, Bioss), p-P70S6K (#97,596, CST, MA, USA), P70S6K (#34,475, CST), NLRP3 (ab263899, Abcam), SOCS3 (bs-24250R, Bioss), TSG101 (bs-1365R, Bioss), CD63 (bs-23032R, Bioss), calnexin (bsm-52639R, Bioss), and β-actin (bs-0061R, Bioss) at 4 °C overnight, followed by application with Goat Anti-Rabbit IgG (ab672, Abcam) for 1 h. The membranes were developed using an enhanced chemiluminescence solution (Yeasen), and the band intensity was quantified by Image J software.

### Extraction and identification of exosomes (Exos)

Exos were extracted from the serum samples of rats using the miRNeasy Mini Kit (Qiagen, CA, USA) following the protocol. Transmission electron microscopy (TEM) was adopted to observe the exosome morphology. The size distribution of the isolated Exos was analyzed by Nanoparticle Tracking Analysis using the Nano Sight NS300 (Malvern Instruments, Malvern, UK). Moreover, specific markers for exosomes (CD63 and TSG101) and endoplasmic reticulum marker (calnexin) were detected by western blotting.

### Isolation of primary ovarian granular cells (GCs) and treatment

Female Sprague–Dawley rats (21–29 days old) were intraperitoneal injected with pregnant mare serum gonadotropin (40 IU). Two days later, the rats were anesthetized by sodium pentobarbital and the ovaries were collected. Using a stereoscopic microscope, the follicles were punctured by a needle to release ovarian GCs. After centrifugation at 500 g for 5 min, the GCs were cultured in DMEM/F-12 (Thermo Fisher) containing 10% fetal bovine serum (Thermo Fisher) at 37 °C with 5% CO_2_. The primary GCs were exposed to LPS (0.5, 1, 5, 10 ug/mL) for 48 h with or without co-culture with Exos.

### Agomir/antagomir treatment and lentivirus infection

miR-30a-5p agomir/antagomir, negative control (NC) agomir/antagomir, and lentiviruses carrying SOCS3 overexpression plasmid (lv- SOCS3) or a scramble RNA (lv-NC) were obtained from Genepharma (Shanghai, China). For cell transfection, the primary GCs were transfected with the above RNAs using Lipofectamine 2000 (Invitrogen) according to manufacturer's protocol.

### Cell counting kit-8 (CCK-8)

The primary GCs were planted into 96-well plates (1 × 10^4^/well) and subjected to various treatments. Subsequently, 10 μL of CCK-8 reagent (Beyotime) was added to wells and reacted for 4 h. The results were read at OD 450 nm on a microplate reader (DeTie, Nanjing, China).

### Pyroptosis detection

The percentage of pyroptotic cells was determined by flow cytometry. After indicated treatment, about 2 × 10^5^ cells were harvested and washed with PBS, followed by incubation with 25% mouse serum for 20 min. Next, cells were washed and incubated with anti-caspase 1 antibody (SU40-07, Invitrogen) at 4 ℃ for 20 min. The cells were stained with propidium iodide (Abcam) for 5 min. The stained GCs were measured on a flow cytometer (Thermo Fisher).

### Immunofluorescence staining

The expression of LC3B in primary GCs was evaluated by immunofluorescence staining. In short, GCs mounted on slides were retrieved, and treated with 5% bovine serum albumin for 30 min. Subsequently, cells were incubated with primary antibody LC3B (PA1-46,286, Thermo Fisher) at 4 °C overnight. Then, the GCs were incubated with the Goat Anti-Rabbit IgG H&L (FITC) (ab6717, 1:1000, Abcam) for 2 h at 37 °C, followed by staining with 4′,6-diamidino-2-phenylindole (DAPI, Boster, Beijing, China) for 5 min. Under a fluorescence microscope, the GCs were observed and photographed.

### RNA immunoprecipitation (RIP)

The interaction between miR-30a-5p and SOCS3 was verified by RNA assay using the Imprint RNA Immunoprecipitation Kit (Sigma‐Aldrich, MO, USA). Briefly, the lysates of primary GCs were incubated with protein A magnetic beads that were ligated with anti‐IgG antibody (ab172730, Abcam) or anti‐AgO2 antibody (ab18673, 1:50, Abcam) at 4℃ overnight. The enrichment of miR-30a-5p and SOCS3 in the immuno-precipitates was assessed by RT‐qPCR.

### Dual-luciferase reporter assay

The wild type (WT) or mutant (MUT) sequences of SOCS3 3'-UTR containing miR-30a-5p binding sites were inserted into pmirGLO plasmid (Promega, WI, USA). The 293 T cells were transfected with SOCS3-WT or SOCS3-MUT together with miR‐30a-5p agomir or NC agomir using Lipofectamine™ 2000 (Thermo Fisher). After transfection for 48 h, the luciferase activities were measured using the Renilla-Firefly Luciferase Dual Assay Kit (Promega).

### Statistical analysis

Data are expressed as the mean ± standard deviation (SD). Statistical significances were evaluated by Student’s t-test for two groups, and a one-way analysis of variance with Tukey’s post-hoc test for multiple groups using SPSS 22.0. *p* < 0.05 was defined as statistical significance.

## Results

### BSHLD administration improved ovarian function of PCOS rats

**Fig. 1:**
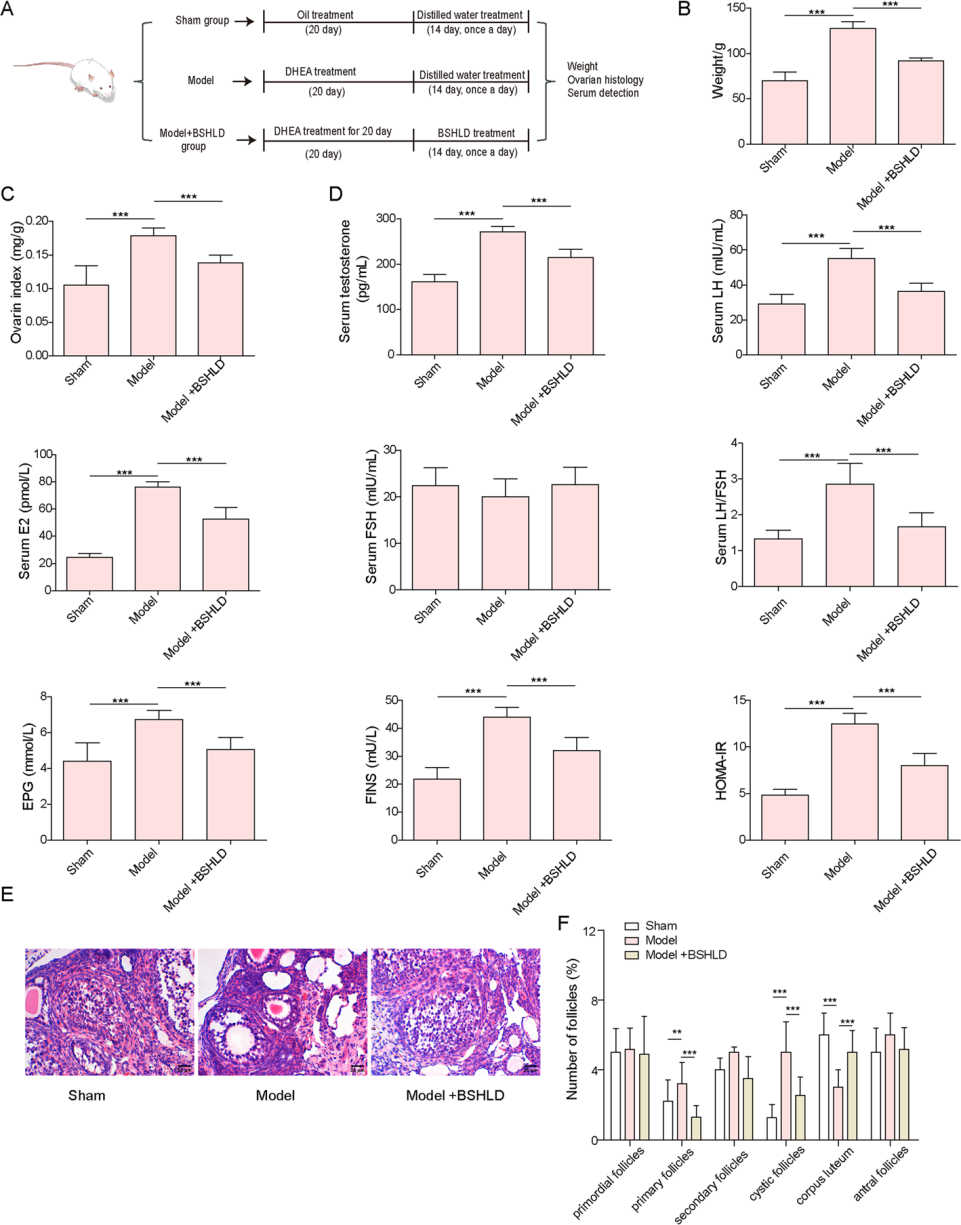
BSHLD administration improved ovarian function of PCOS rats. **A** The schematic diagram of animal experiment procedure. **B-C** The measurement of body weight and ovarian index of rats. **D** The assessment of serum levels of testosterone, LH, E2, FSH, FPG and FINS, LH/FSH ratio, and HOMA-IR value of each group. **E** HE staining was performed to observe pathological changes in ovarian tissues of rats. **F** The number of primordial, primary, secondary, cystic follicles, antral follicles, and corpus luteum of rats were recorded. Results are presented as mean ± SD. *n* = 10. * *p* < 0.05, ** *p* < 0.01, *** *p* < 0.001

The PCOS rat model was established by injection with DHEA, and the regulatory roles of BSHLD on ovarian function were investigated. The experimental schedule is shown in Fig. 1A. In comparison with the sham group, an obvious elevation in body weight and ovarian index was found in PCOS rats, which were weakened by BSHLD treatment (Fig. 1B&C). In addition, PCOS rats exhibited higher serum levels of testosterone, LH, E2, EPG, FINS, lower serum level of FSH, and increased LH/FSH ratio and HOMA-IR value as compared with sham rats; however, BSHLD effectively counteracted these changes (Fig. 1D). Furthermore, HE staining showed a normal ovarian morphology of sham rats as confirmed by intact and regularly arranged granulosa cells, and the presence of corpus luteum and follicles, while multiple follicles, and inflammatory infiltration were observed in PCOS rats (Fig. 1E). These pathological changes in ovarian tissues of PCOS rats were notably attenuated by BSHLD (Fig. 1E). Moreover, compared with sham group, the number of primary follicles and cystic follicles in PCOS rats were increased significantly, while the number of corpus luteum was decreased. After BSHLD treatment, the number of primary follicles and cystic follicles were reduced, while corpus luteum number was elevated. In addition, there were no significant difference in primordial follicles, secondary follicles, and antral follicles among groups (Fig. 1F). These results suggested that BSHLD alleviated ovarian pathological changes and dysfunction in PCOS rats.

### BSHLD repressed autophagy and NLRP3-mediated pyroptosis in PCOS rats

**Fig. 2:**
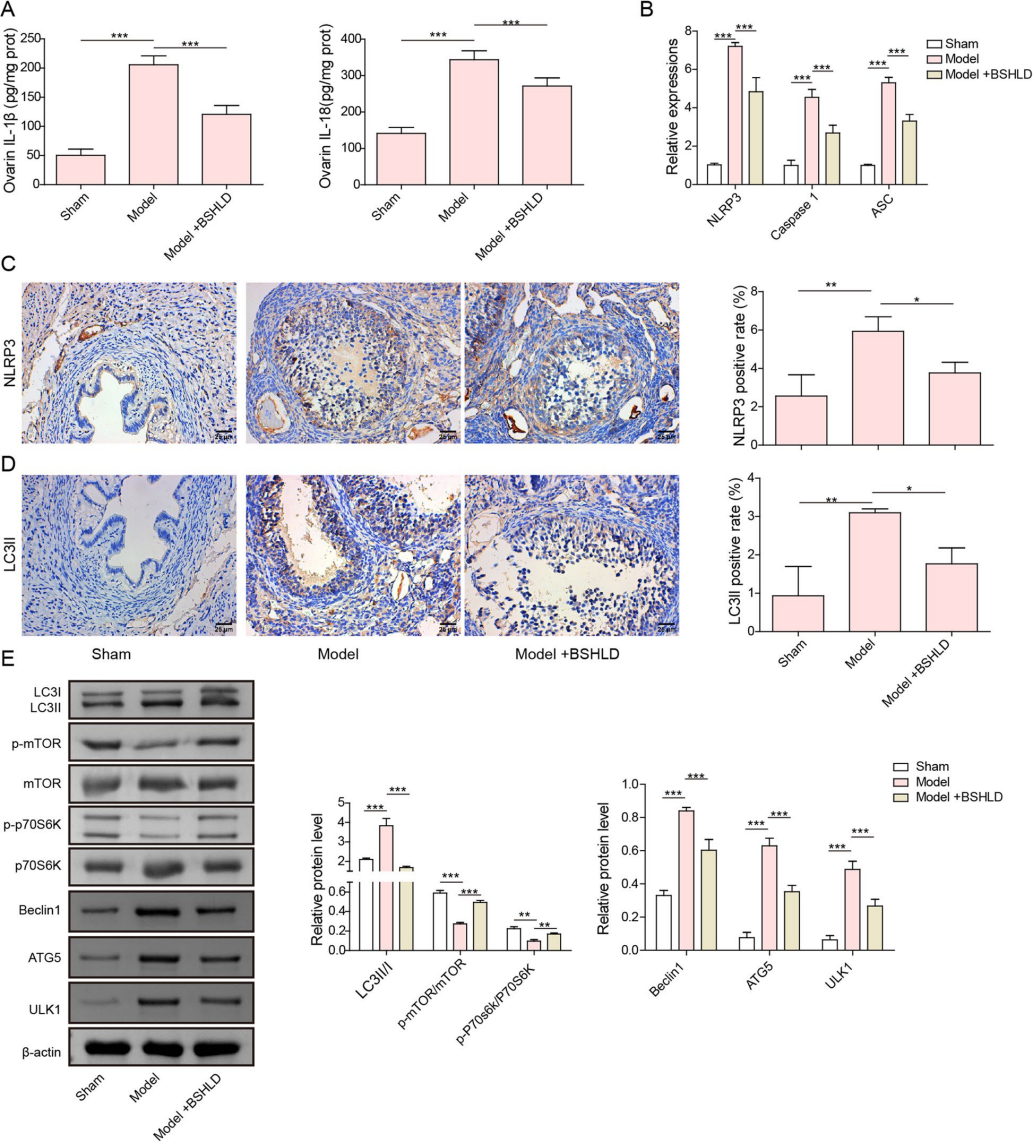
BSHLD repressed autophagy and NLRP3-mediated pyroptosis in PCOS rats. **A** ELISA assay for the evaluation of IL-1β and IL-18 levels in ovarian tissues. **B** RT-qPCR analysis of NLRP3, ASC, and Caspase 1 mRNA levels. **C-D** Immunohistochemical staining examined the expression of NLRP3 and LC3B. **E** Western blotting analysis was performed for the quantification of ATG5, Beclin1, ULK1, LC3II/I, p-mTOR, mTOR, p-P70S6K, and P70S6K protein levels. Results are presented as mean ± SD. *n* = 10. * *p* < 0.05, ** *p* < 0.01, *** *p* < 0.001

Pyroptosis and autophagy have been verified to be implicated in PCOS pathogenesis [27, 28]. We further evaluated whether BSHLD exerted its beneficial effect through modulation of pyroptosis and autophagy. ELISA assay discovered that the levels of IL-1β and IL-18 in ovarian tissues of PCOS rats were significantly elevated as compared to sham group, while BSHLD administration greatly reversed IL-1β and IL-18 production (Fig. 2A). Moreover, RT-qPCR analysis showed that NLRP3, ASC, and Caspase 1 mRNA levels were up-regulated in ovarian tissues of PCOS rats, and their expression levels were reversed by BSHLD treatment (Fig. 2B). Both immunohistochemical staining and western blotting results indicated that the protein levels of NLRP3, ATG5, Beclin1, ULK1, as well as LC3II/I radio were enhanced, while the phosphorylation of mTOR and P70S6K was suppressed in ovarian tissues of PCOS rats, whereas BSHLD intervention abolished the above alterations (Fig. 2C, D, E). Thus, NLRP3-mediated pyroptosis and excessive autophagy in ovarian tissues of PCOS rats could be repressed by BSHLD treatment.

### BSHLD reduced miR-30a-5p expression in serum, serum-Exos and ovarian tissues in PCOS rats

**Fig. 3:**
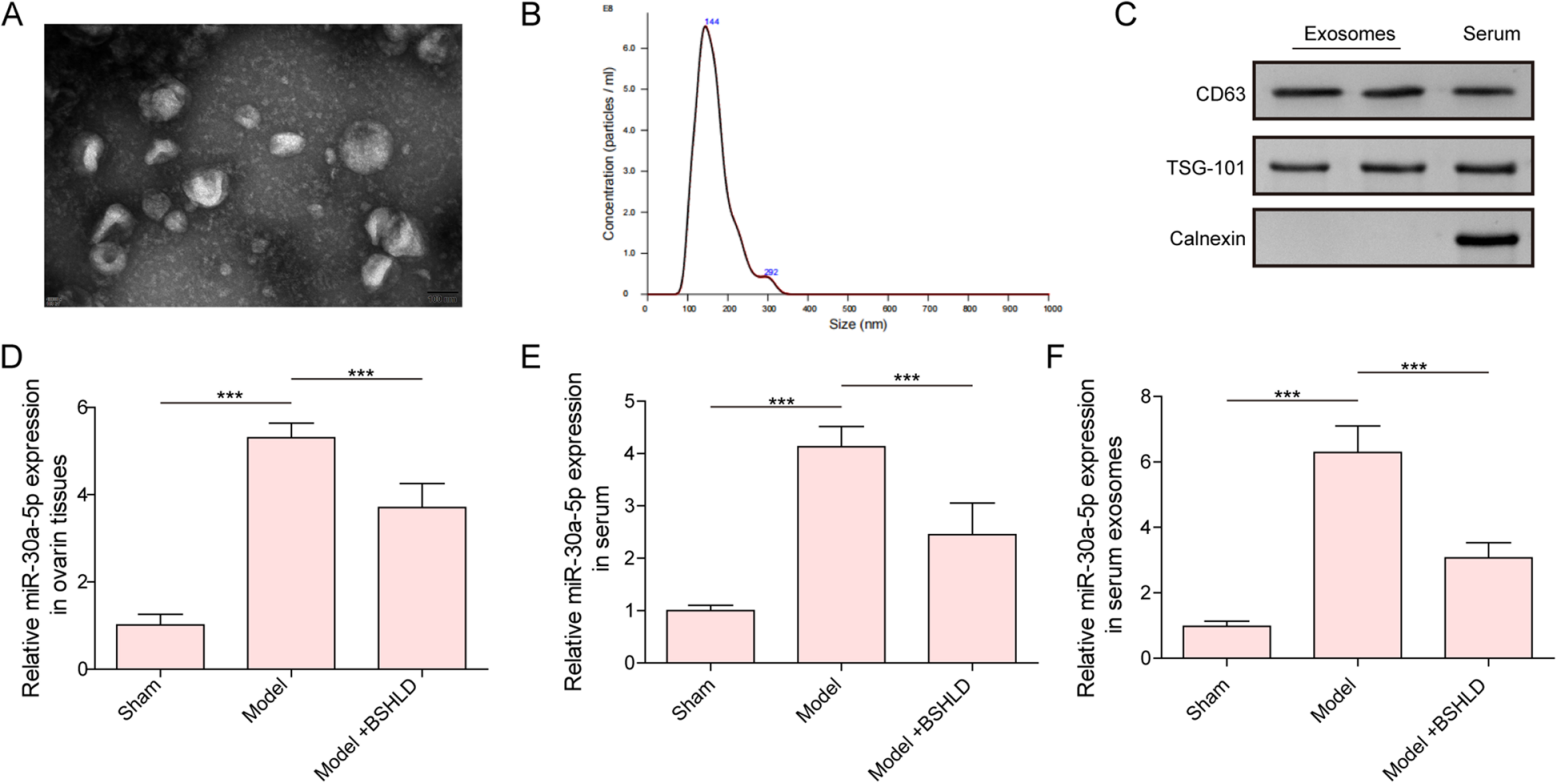
BSHLD reduced miR-30a-5p expression in serum, serum-exos and ovarian tissues in PCOS rats. **A** The microstructure of Exos was observed by TEM. **B** Nanoparticle Tracking Analysis evaluated the size distribution of Exos. **C** Western blotting analysis of TSG101, CD63, and calnexin protein levels. **D-F** Expression of miR-30a-5p in ovarian tissues, serum, and serum-Exos was assessed by RT-qPCR. Results are presented as mean ± SD. *n* = 10. * *p* < 0.05, ** *p* < 0.01, *** *p* < 0.001

We further isolated serum-derived Exos from rats. The extracted Exos with bilayer vesicles were observed by TEM (Fig. 3A). Nanoparticle tracking analysis validated the serum-derived Exos with a size distribution of 30-150 nm (Fig. 3B). Additionally, the presence of TSG101 and CD63, but the absence of calnexin were validated in Exos (Fig. 3C), suggesting the successful isolation of Exos. Notably, miR-30a-5p expression was evidently raised in the ovarian tissues, serum, and serum-derived Exos of PCOS rats, whereas BSHLD treatment partly reversed the increased level of miR-30a-5p (Fig. 3D-F). The above findings demonstrated that BSHLD treatment lowered serum-derived exsomal miR-30a-5p level in PCOS rats.

###  BSHLD inhibited LPS-induced autophagy in GCs via reducing exosomal miR-30a-5p

**Fig. 4:**
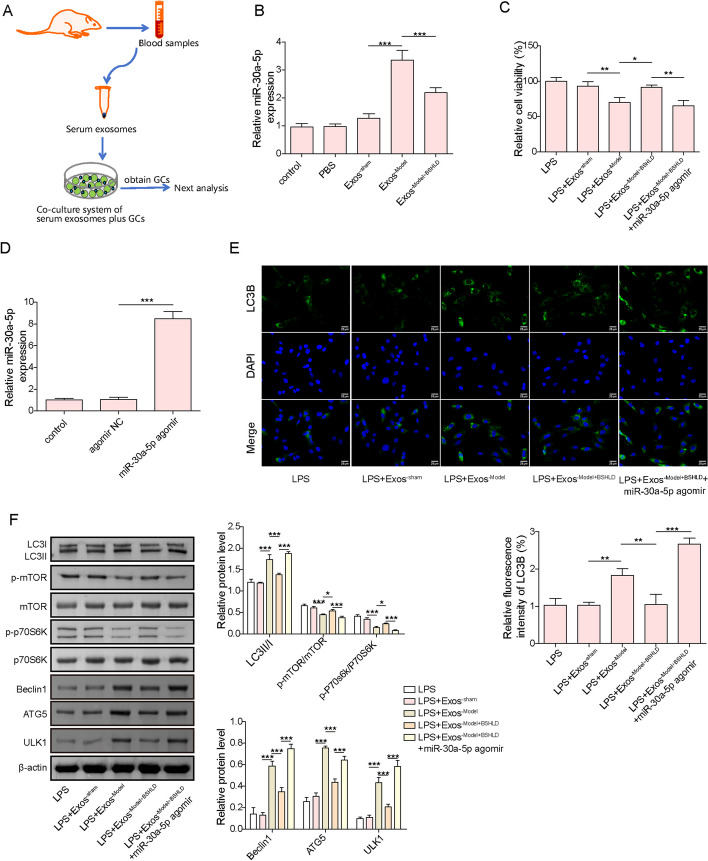
BSHLD inhibited LPS-induced autophagy in GCs via reducing exosomal miR-30a-5p. Primary GCs were co-cultured with PBS or Exos derived from serum samples of sham, Model and BSHLD group rats. **A** The schematic diagram of the co-culture system of serum-derived Exos and GCs. **B** Expression of miR-30a-5p in GCs was measured by RT-qPCR. **C** Primary GCs were treated with miR‐30a-5p agomir or agomir NC for 48 h, and the expression of miR-30a-5p was assessed by RT-qPCR. **D** The viability of GCs was determined by CCK-8. **E** LC3B expression in GCs was evaluated by immunofluorescence staining. **F** The protein levels of ATG5, Beclin1, ULK1, LC3II/I, p-mTOR, mTOR, p-P70S6K, and P70S6K in GCs were evaluated by Western blotting. Results are presented as mean ± SD. *n* = 3. * *p* < 0.05, ** p < 0.01, *** *p* < 0.001

To verify whether BSHLD restrained autophagy by modulation of exosomal miR-30a-5p, primary ovarian GCs were isolated and co-cultured with serum-derived Exos from different groups (Fig. 4A). First, the expression of miR-30a-5p in GCs was quantified by RT-qPCR. The expression of miR-30a-5p in primary ovarian GCs treated with Exos^−sham^ miR-30a-5p expression at a certain extent, while there was no significant difference, however, Exos^−model^ administration greatly enhanced miR-30a-5p expression in GCs, while this effect was partly diminished upon BSHLD treatment (Fig. 4B). Moreover, CCK-8 showed that the viability of GCs was not affected by Exos^−Sham^, but markedly reduced by Exos^−model^, and the effect of Exos^−model^ could be abolished by BSHLD treatment^.^ (Supplementary Fig. S1). Moreover, after exposure to LPS, and the viability of GCs was declined in a concentration depend manner (supplementary Fig. S2A), while miR-30a-5p level was raised in a dose dependent manner (supplementary Fig. S2B). Next, miR-30a-5p was overexpressed in GCs by transfection with miR-30a-5p agomir, and its overexpression efficiency was validated by RT-qPCR (Fig. 4C). In LPS-treated GCs, the inhibitory role of Exo^−Model^ administration in cell viability was reversed by Exos^−model+BSHLD^ treatment, however, the cell viability was further suppressed upon miR-30a-5p was overexpressed (Fig. 4D). In addition, Exos^−model^-induced the increase of ATG5, Beclin1, ULK1, and LC3II/I levels and the decrease of p-mTOR, and p-P70S6K were prevented by Exos^−model+BSHLD^, while co-treatment with miR-30a-5p agomir remarkably diminished the effects of Exos^−model+BSHLD^ (Fig. 4E&F). These observations suggested that LPS-induced excessive autophagy was suppressed by BSHLD via inhibiting serum exosomal miR-30a-5p.

### BSHLD attenuated LPS-triggered pyroptosis in GCs by decreasing exosomal miR-30a-5p

**Fig. 5:**
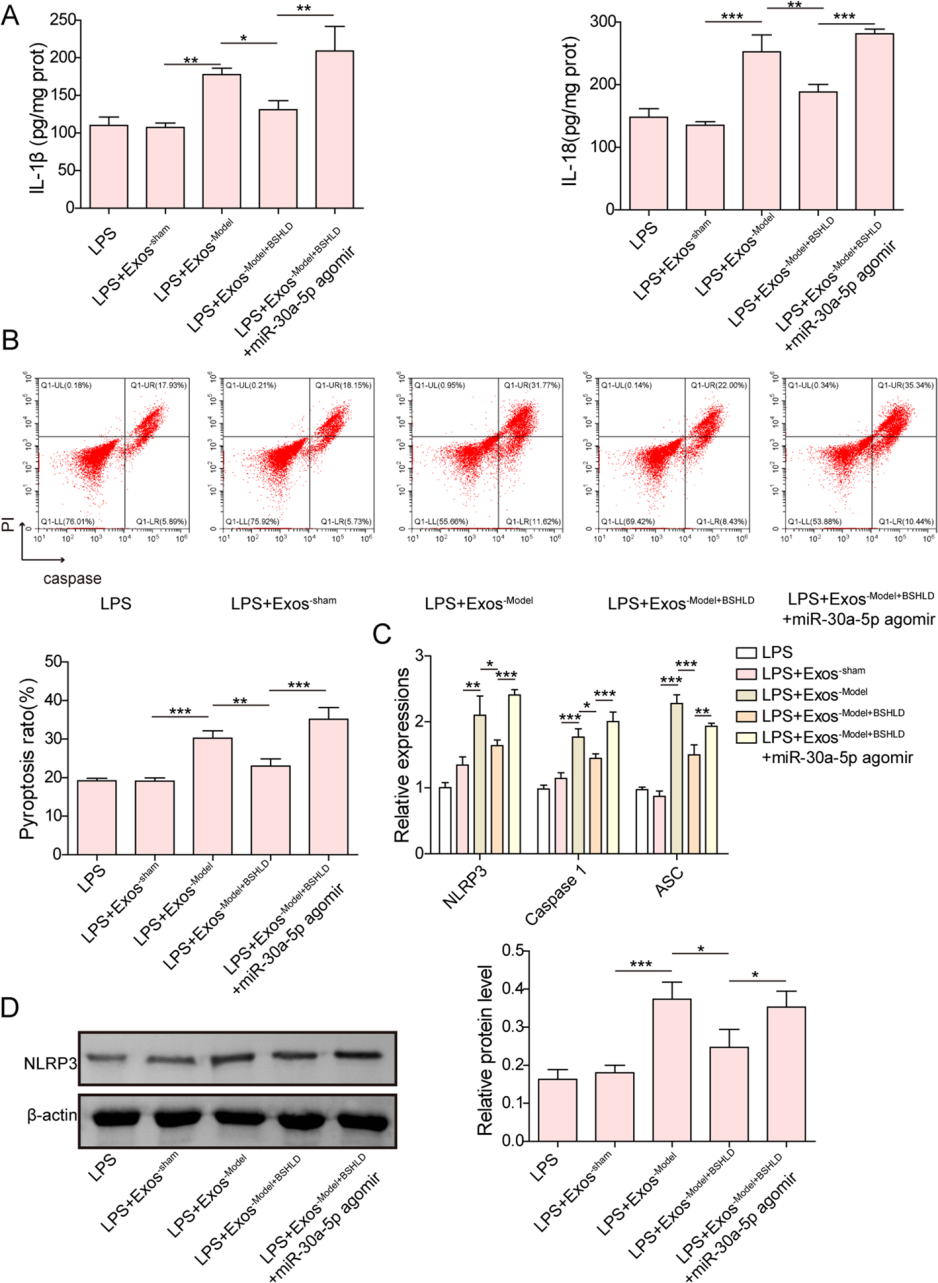
BSHLD attenuated LPS-triggered pyroptosis in GCs by decreasing exosomal miR-30a-5p. **A** ELISA analysis of IL-1β and IL-18 levels in the supernatant of GCs. **B** The pyroptosis of GCs was determined by flow cytometry. **C** RT-qPCR analysis of NLRP3, ASC, and Caspase 1 mRNA levels in GCs. **D** The protein abundance of NLRP3 was detected by Western blotting. Results are presented as mean ± SD. *n* = 3. * *p* < 0.05, ** *p* < 0.01, *** *p* < 0.001

The influence of BSHLD-modulated exosomal miR-30a-5p on ovarian granular cell pyroptosis was further investigated. The release of IL-1β and IL-18 from LPS-challenged GCs was enhanced by Exos^−Model^, which was inhibited by Exos^−Model+BSHLD^ (Fig. 5A). However, overexpression of miR-30a-5p eliminated Exos^−Model+BSHLD^ -mediated inhibition in IL-1β and IL-18 production (Fig. 5A). Besides, Exos^−Model+BSHLD^ could abolish the promotive role of Exos^−Model^ in pyroptosis of GCs, and this role of Exos^−Model+BSHLD^ was counteracted by co-treatment with miR-30a-5p agomir (Fig. 5B). As compared to Exos^−sham^ group, Exos^−Model^ treatment significantly increased the levels of NLRP3, ASC, and Caspase 1, while these effects were impeded by Exos^−Model+BSHLD^ (Fig. 5C&D). Similarly, the repressive roles of Exos^−Model+BSHLD^ on pyroptosis-related marker levels were reversed by miR-30a-5p overexpression (Fig. 5C&D). To sum up, BSHLD suppressed NLRP3-mediated pyroptosis of GCs through down-regulating exosomal miR-30a-5p.

### miR-30a-5p directly targeted SOCS3 to inactivate mTOR/P70S6K signaling

**Fig. 6:**
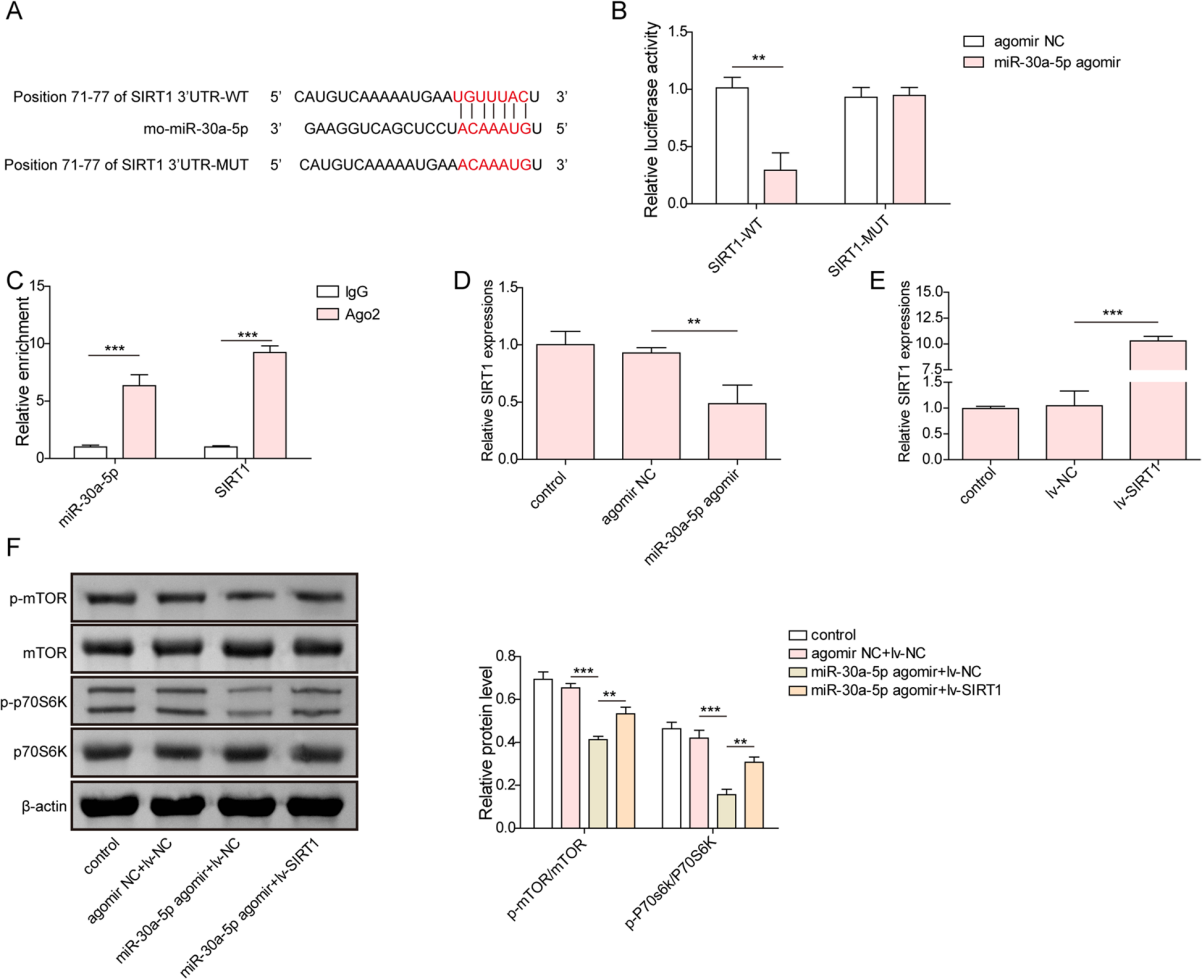
miR-30a-5p directly targeted SOCS3 to inactivate mTOR/P70S6K signaling. **A** The binding sites of miR-30a-5p on SOCS3 3’UTR were predicted by TargetScan database. **B-C** The direct binding of miR-30a-5p to SOCS3 was validated by dual luciferase reporter assay and RIP assay. **D** RT-qPCR analysis of SOCS3 level in GCs treated with miR‐30a-5p agomir or agomir NC. **E** The expression of SOCS3 was detected by RT-qPCR after transfection with lv-NC or lv- SOCS3. **F** Western blotting analysis of p-mTOR, mTOR, p-P70S6K, and P70S6K levels in GCs. Results are presented as mean ± SD. *n* = 3. * *p* < 0.05, ** *p* < 0.01, *** *p* < 0.001

As predicted by bioinformatics analysis, miR-30a-5p could bind to the 3’-UTR sequences of SOCS3 (Fig. 6A). Then, the direct interplay between miR-30a-5p and SOCS3 was validated by dual luciferase reporter and RIP assays. As shown in Fig. 6B, in comparison with agomir NC group, miR-30a-5p agomir dramatically suppressed the luciferase activity of SOCS3-WT transfected cells, while miR-30a-5p agomir did not affect the luciferase activity of SOCS3-MUT group. Moreover, both miR-30a-5p and SOCS3 showed high abundance in precipitated complex by AgO2 antibody (Fig. 6C). RT-qPCR discovered that overexpression of miR-30a-5p remarkably decreased SOCS3 expression (Fig. 6D). Next, SOCS3 expression was greatly enhanced by infection with lentiviruses carrying SOCS3-overexpressing plasmid (Fig. 6E). Subsequently, western blotting revealed that miR-30a-5p overexpression obviously reduced p-mTOR, and p-P70S6K protein levels in GCs, whereas SOCS3 overexpression abolished the above alterations (Fig. 6F). Therefore, miR-30a-5p caused inactivation of mTOR/P70S6K pathway via targeting SOCS3.

### Rapamycin reversed miR-30a-5p antagomir-mediated inhibitory role on autophagy and pyroptosis of GCs

**Fig. 7:**
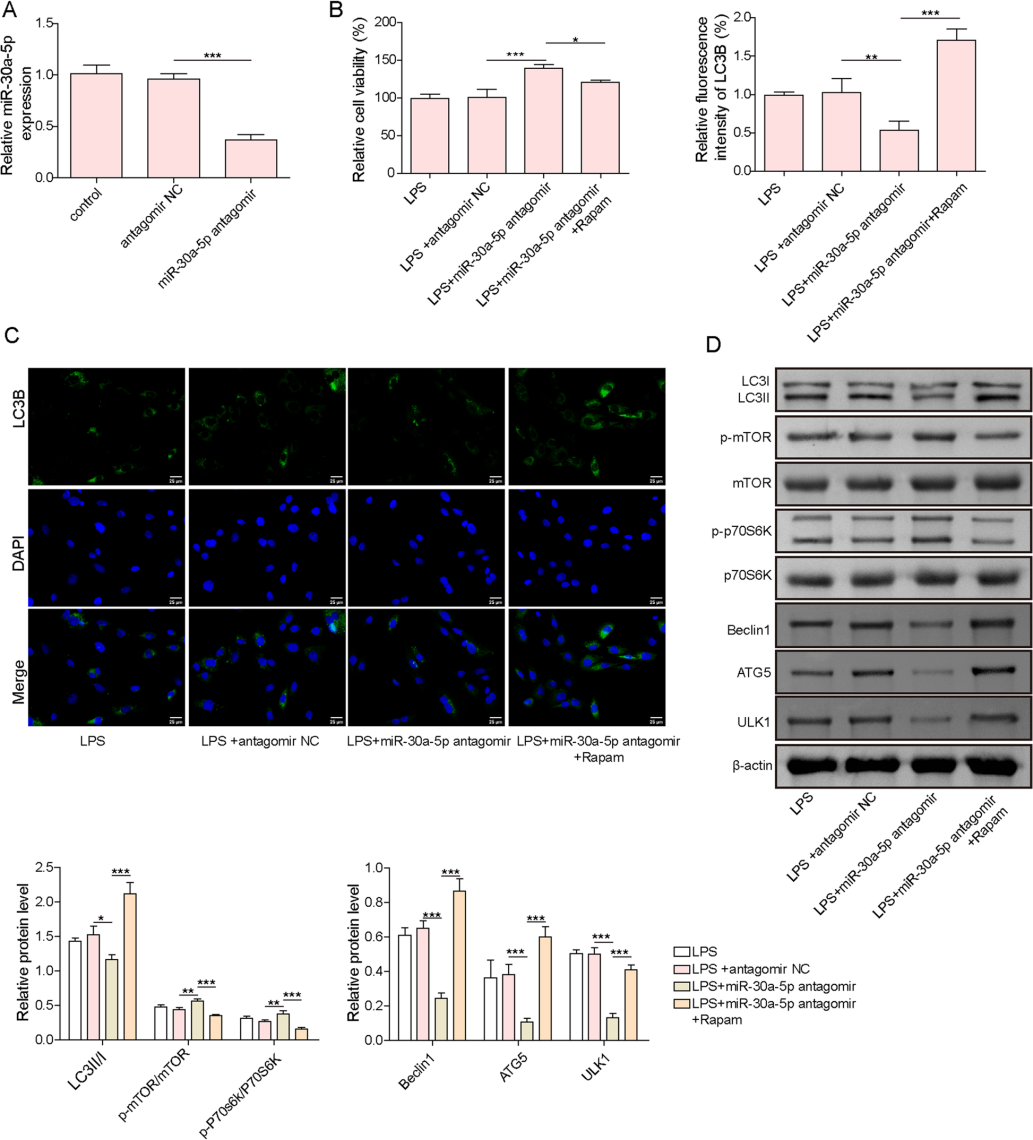
Rapamycin reversed miR-30a-5p antagomir-mediated inhibitory role in GCs autophagy. **A** RT-qPCR analysis of miR‐30a-5p expression in GCs after treatment with miR‐30a-5p antagomir or NC antagomir. GCs were treated with miR-30a-5p antagomir alone or co-treated with miR-30a-5p antagomir + Rapamycin, and followed by exposure to LPS. **B** The viability of GCs was detected by CCK-8. **C** LC3B expression in GCs was observed by immunofluorescence staining. **D** The protein abundance of ATG5, Beclin1, ULK1, LC3II/I, p-mTOR, mTOR, p-P70S6K, and P70S6K in GCs was measured by Western blotting. Results are presented as mean ± SD. *n* = 3. * *p* < 0.05, ** *p* < 0.01, *** *p* < 0.001

**Fig. 8:**
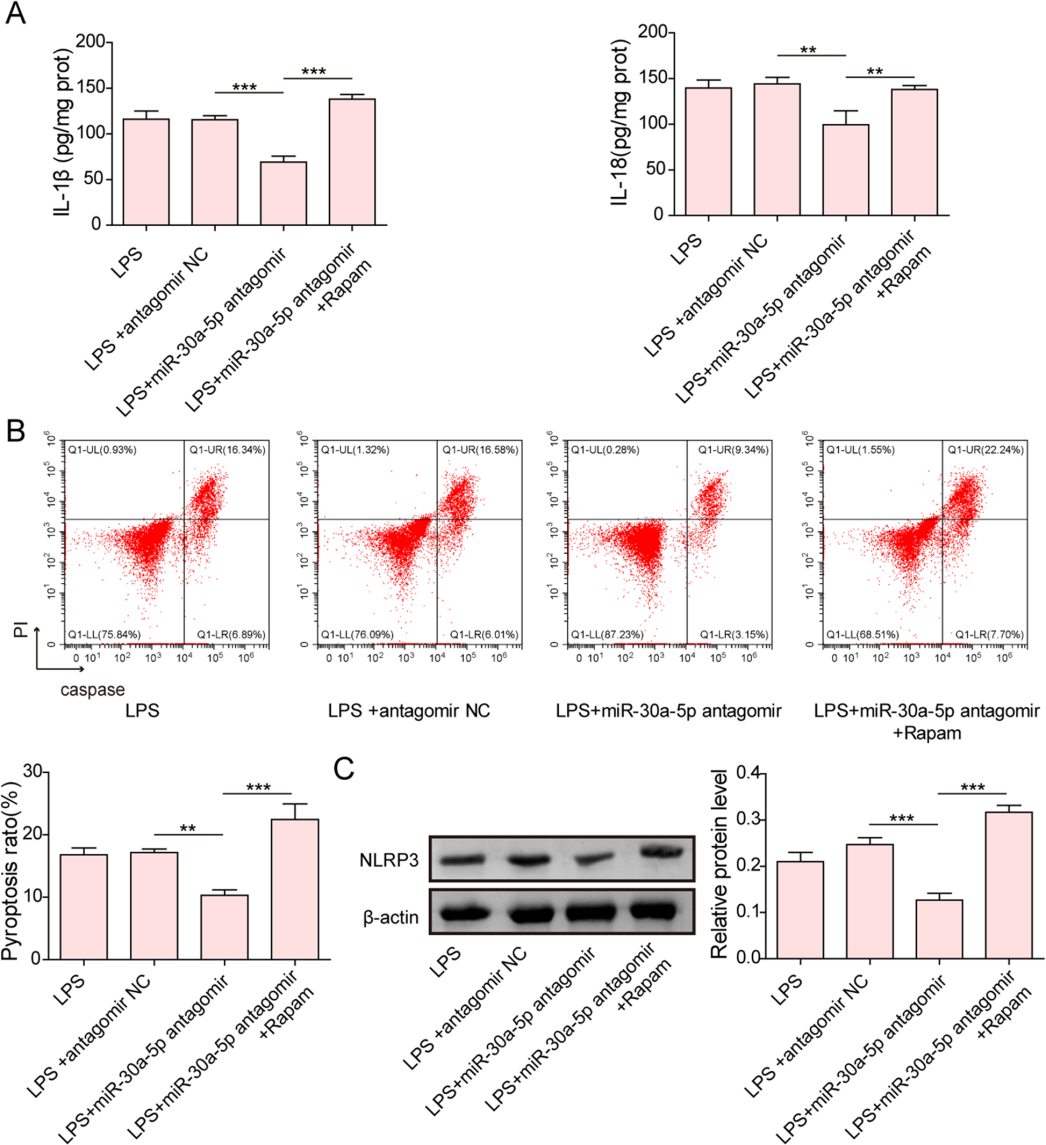
Rapamycin reversed miR-30a-5p antagomir-mediated inhibitory role on GCs pyroptosis. GCs were treated with miR-30a-5p antagomir alone or co-treated with miR-30a-5p antagomir + Rapamycin, and followed by exposure to LPS. **A** The release of IL-1β and IL-18 from GCs was evaluated by ELISA. **B** The percentage of pyroptotic GCs was detected by flow cytometry. **C** Western blotting measured the protein level of NLRP3. Results are presented as mean ± SD. *n* = 3. * *p* < 0.05, ** *p* < 0.01, *** *p* < 0.001

We further validated whether mTOR/P70S6K pathway was involved in miR-30a-5p-mediated regulation in autophagy and pyroptosis of GCs. For this purpose, GCs were transfected with miR-30a-5p antagomir, followed by treatment with rapamycin (mTOR inhibitor). RT-qPCR assay showed that the expression of miR-30a-5p was strikingly declined after treatment with miR-30a-5p antagomir (Fig. 7A). It was observed that the viability of LPS-treated GCs was enhanced by miR-30a-5p inhibition, whereas rapamycin co-treatment greatly abolished the role of miR-30a-5p inhibition (Fig. 7B). Immunofluorescence and western blotting assays suggested that the levels of ATG5, Beclin1, ULK1, and LC3II/I radio were declined and the phosphorylation of mTOR and p70S6K were enhanced by miR-30a-5p down-regulation in LPS-treated GCs, and these changes were markedly diminished after rapamycin administration (Fig. 7C&D). Similar trends were observed in GC pyroptosis, miR-30a-5p knockdown strikingly inhibited IL-1β and IL-18 release, reduced pyroptotic cell rate and NLRP3 expression in LPS-exposed GCs, and all these effects were abrogated by rapamycin co-treatment (Fig. 8A-C). Taken together, miR-30a-5p inhibition repressed autophagy and pyroptosis of GCs via inactivation of mTOR/P70S6K pathway.

## Discussion

PCOS is a heterogeneous endocrine disease featured by ovarian dysfunction, androgen excess and polycystic ovarian change. Currently, TCM possesses distinct advantages such as multiple targets and fewer side effects, which is widely applied to treat PCOS in clinical practice [29]. In this investigation, we aimed to uncover the therapeutic effects and possible mechanism of BSHLD in PCOS. The results showed that BSHLD improved ovarian function and polycystic ovarian morphology of PCOS rats. Mechanistically, BSHLD reduced exosomal miR-30a-5p level, which inactivated mTOR/P70S6K pathway via targeting SOCS3, thereby repressing excessive autophagy and pyroptosis to alleviate PCOS. Our findings provided evidence for the clinical application of BSHLD in treating PCOS.

So far, letrozole or other medicines are first-line treatments for PCOS. However, their side effects greatly lower patients’ compliance [30]. Chinese herbal medicines have been widely used to treat PCOS due to their fewer side effects and adverse reactions during long-term treatment [31], supporting the advantage of Chinese herbal medicines. Particularly, Yinyanghuo, one composition of BSHLD, could improve PCOS symptoms in rats [32]. Chuanxiong, another drug in BSHLD, was reported to restrain pyroptosis, thereby improving inflammatory state in diabetic nephropathy model [33]. Lactone component exacted from Chuanxiong that is a composition of BSHLD, has been suggested to mitigate myocardial ischemia injury by suppressing autophagy [34]. All these findings from previous studies implied the therapeutic potential of BSHLD in the treatment of PCOS. In this study, we provided first evidence that BSHLD improved PCOS through restraining the hyperactivated autophagy and NLRP3-mediated pyroptosis. Therefore, our findings suggested Chinese herbal medicine BSHLD as an effective alternative drug for PCOS.

Although the therapeutic effects of BSHLD have been confirmed, its potential mechanism remains largely unknown. In the present work, we further studied the potential mechanism of BSHLD underlying PCOS treatment. Exosomal cargos such as miRNAs have been recognized as biomarkers for PCOS [35]. A series of plasma exosomal microRNAs have been identified to be differentially expressed in PCOS patients, which exhibited close association with PCOS progression [36]. Gao et al. documented that exosomal miR-143-3p/miR-155-5p facilitated glycolytic activation of GCs, which resulted in follicular dysplasia in PCOS [37]. miR-18b-5p, derived from follicular fluid exosomes, was found to delay PCOS progression via targeting PTEN to activate PI3K/Akt/mTOR signaling [38]. miR-30a-5p is a member of miR-30 family. Up-regulation of miR-30a-5p has been validated in clinical PCOS patients [39] and experimental PCOS model [40]. Consistently, our preliminary experiments found that miR-30a-5p was highly expressed in the serum, serum-derived Exos, and ovarian tissues of PCOS rats, whereas BSHLD administration evidently reduced miR-30a-5p expression. It has been reported that miR-30a-5p is crucially involved in various pathophysiological processes. For example, miR-30a-5p was reported to be significantly correlated with insulin resistance in patients with Alstrőm and Bardet-Biedl syndromes [41]. Moreover, miR-30a-5p was responsible for inflammation and osteogenesis inhibition in periodontitis [42], and could drive NF-kB/NLRP3 signaling pathway to accelerate chronic heart failure [43]. It was also confirmed that miR-30a-5p could promote autophagy during porcine circovirus type 2 infection [44]. Notably, both insulin resistance, autophagy activation and NLRP3-meidated inflammation were critical risk factor of PCOS. NLRP3 inflammasome has been reported to act as a sensor of pyroptosis. Pyroptosis is a novel type of inflammation-related programmed cell death associated with inflammasome formation [45]. Mounting evidence has suggested that pyroptosis is involved in the pathogenesis of PCOS [46, 47]. The activated NLRP3 inflammasomes recruits ASC to cleave and activate caspase 1, which subsequently facilitates the maturation of proinflammatory cytokines, including IL-1β and IL-18 [48]. Surprisingly, our results also found that the decreased expression of miR-30a-5p in exosomes caused by BSHLD was accompanied by decreased levels of GCs autophagy and NLRP3-mediated pyroptosis, as evidenced by reducing NLRP3, caspase-3, ASC, IL-1β and IL-18 levels, whiles these changes were all markedly reversed after miR-30a-5p overexpression, which implied that exosomal miR-30a-5p might be one of the markers of disease progression of PCOS. Overall, these findings further supported the pathogenic function of miR-30a-5p in vitro, and suggested that exosomal miR-30a-5p was involved in the therapeutic mechanism of BSHLD in PCOS.

SOCS family consists of eight members: SOCS1 through SOCS7 and the cytokine-inducible Src homology 2 domain-containing protein (CIS). It was well known that SOCS family members were capable to regulate various of signaling pathway, such as Janus kinases (JAKs)/ signal transducer and activator of transcription (STAT)-, nuclear factor kB (NF-kB)-, Akt/mTOR-mediated pathways [49]. SOCS3 is one of the negative regulators of cytokine signaling that function through STAT3 pathway. SOCS3, an inflammation-related molecule, was proved to be associated with markers of insulin sensitivity and inflammation in PCOS [50]. It has been reported that SOCS3 inhibition could promote ovarian angiogenesis during PCOS by activating STAT3/VEGFA signaling pathway [51]. Kerbus et al.documented that SOCS3 deletion could improve metabolic dysfunction in mice with PCOS [52], implying SOCS3 was a critical pathogenic factor of PCOS. In the present work, SOCS3 was validated as a target gene of miR-30a-5p in GCs, and could reverse miR-30a-5p-mediated GCs pyroptosis and autophagy.

mTOR is a serine/threonine protein kinase that is an inhibitor of autophagy via suppressing autophagosome formation [53]. mTOR/p70S6K pathway has been recognized as a classical autophagy pathway [54]. It has been indicated that activation of the mTOR/p70S6K pathway results in autophagy inhibition [55]. mTOR was found to be involved in the pathogenesis of PCOS via affecting metabolic reprogramming [56]. Modulation of mTOR pathway was found to participate in the protective role of nanocurcumin in insulin resistance and pancreatic deficits in PCOS rats [57]. Shao et al.also disclosed that SOCS3 repressed hepatitis C virus replication in an mTOR-dependent manner [49], further supporting SOCS3 play as a key regulator of mTOR signaling. In the present work, we findings revealed that application of the mTOR agonist rapamycin remarkably reversed miR-30a-5p deficiency-mediated inhibitory effect on excessive autophagy and pyroptosis. These findings suggested that SOCS3 acted as a downstream target of exosomal miR-30a-5p, and participated in BSHLD-mediated protection against PCOS through mTOR/p70S6K pathway, which further enriches our understanding of the pathogenesis of PCOS.

**Fig. 9:**
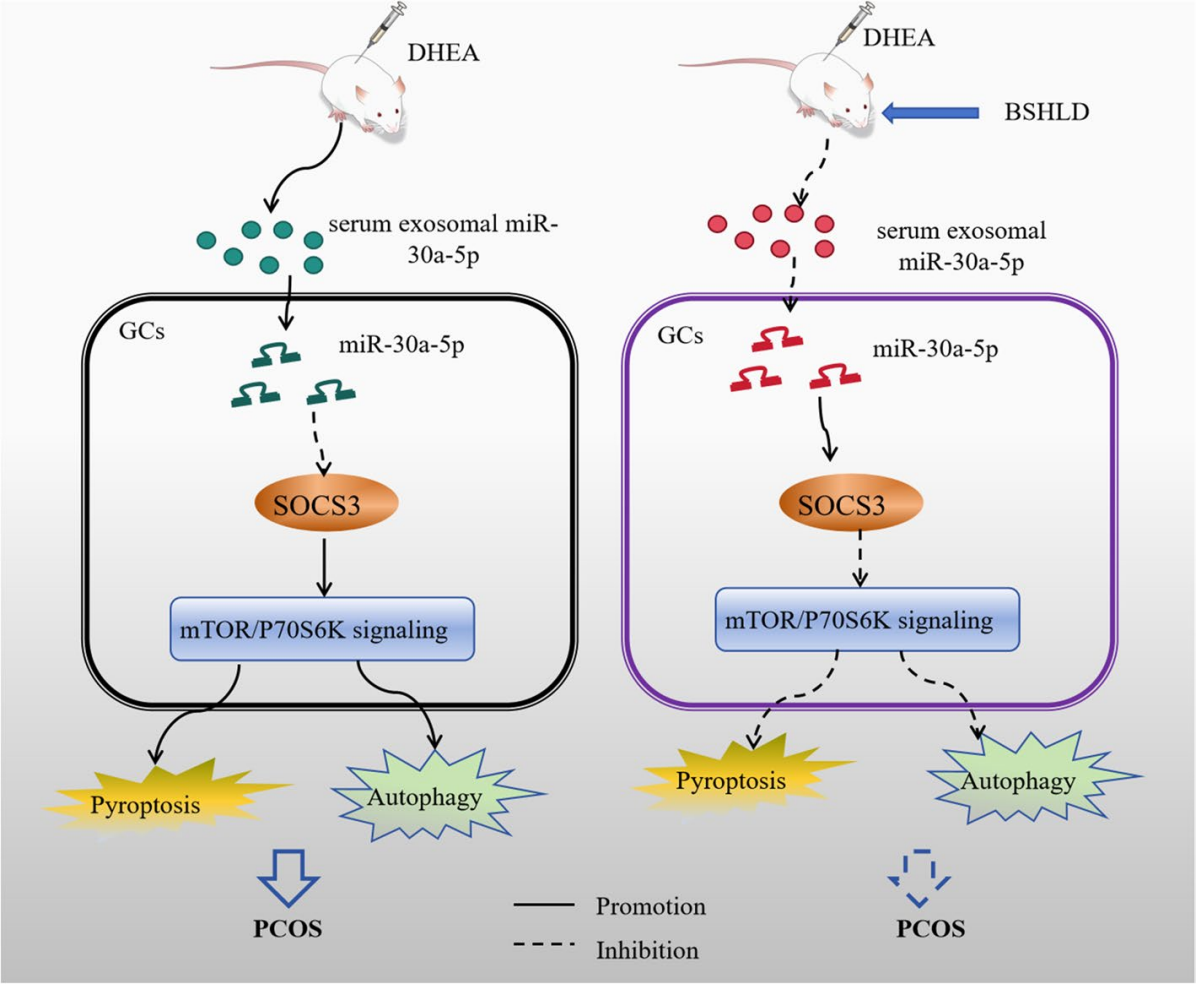
Research mechanism diagram: BSHLD improves PCOS by regulating exosomal miR-30a-5p/ SOCS3/mTOR/NLRP3 signaling-mediated autophagy and pyroptosis

Taken together, the current study validated the BSHLD suppressed exosomal miR-30a-5p expression to activate SOCS3/mTOR/p70S6K signaling, thus inhibiting the excessive autophagy and pyroptosis of GCs (Fig. 9). Our study may provide guiding significance for the formation of a consensus on the TCM replacement therapy for PCOS. Besides, the target molecules of BSHLD, including miR-30a-5p and SOCS3, might be used as the diagnosis and treatment targets of PCOS, which may also enrich the understanding of pathogenesis of this disease.

### Supplementary Information

**Additional file 1:** **Supplementary Fig. 1.** Effect of serum-exos on the viability of GCs. The viability of GCs was determined by CCK-8. Results are presented as mean ± SD. *n* = 3. * *p* < 0.05, ** *p* < 0.01, *** *p* < 0.001.

**Additional file 2:** **Supplementary Fig. 2.** Effect of LPS on the viability and miR-30a-5p expression in GCs. GCs were exposed to LPS (0.5, 1, 5, 10 μg/mL) for 48 h. (A) The viability of GCs was assessed by CCK-8. (B) Expression of miR-30a-5p was detected by RT-qPCR. Results are presented as mean ± SD. *n* = 3. * *p* < 0.05, ** *p* < 0.01, *** *p* < 0.001.

## Data Availability

All data generated or analyzed are included in this article. Further inquiries can be directed to the corresponding author.

## Abbreviations

PCOS: Polycystic ovary syndrome
TCM: Traditional Chinese medicine
BSHLD: Bushenhuoluo Decotion
mTOR: Mechanistic target of rapamycin kinase
p70S6K: P70 Ribosomal protein S6 kinase
miRNAs: MicroRNAs
SOCS3: Suppressor of cytokine signaling 3
DHEA: Dehydroepiandrosterone
E2: Estradiol
LH: Luteinizing hormone
FSH: Follicle-stimulating hormone
FINS: Fasting insulin
FPG: Fasting plasma glucose
HOMA-IR: Homeostasis model assessment of insulin resistance
HE: Hematoxylin-eosin
Exos: Exosomes
TEM: Transmission electron microscopy
GCs: Granular cells
NC: Negative control
CCK-8: Cell counting kit-8
RT-qPCR: Reverse transcription quantitative PCR
DAPI: 4′,6-Diamidino-2-phenylindole
RIP: RNA immunoprecipitation
WT: Wild type
MUT: Mutant
SD: Standard deviation
