# Supplementary figures and images for: Bushenhuoluo Decoction improves polycystic ovary syndrome by regulating exosomal miR-30a-5p/ SOCS3/mTOR/NLRP3 signaling-mediated autophagy and pyroptosis
Source: J Ovarian Res. 2024 Feb 1;17:29. doi: 10.1186/s13048-024-01355-x (PMC10832128; doi:10.1186/s13048-024-01355-x)

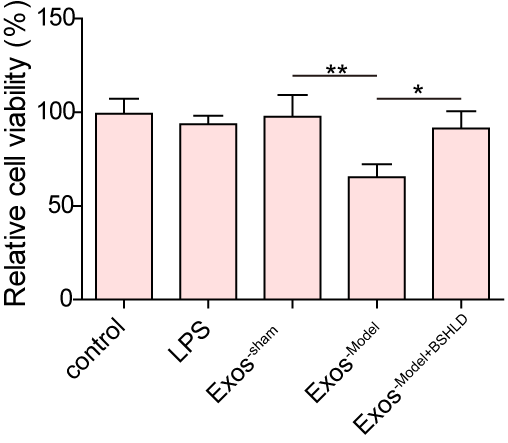

Supplement: Supplementary file 1 — Additional file 1: Supplementary Fig. 1. Effect of serum-exos on the viability of GCs. The viability of GCs was determined by CCK-8. Results are presented as mean ± SD. n = 3. * p < 0.05, ** p < 0.01, *** p < 0.001. [file 13048_2024_1355_MOESM1_ESM.tif]

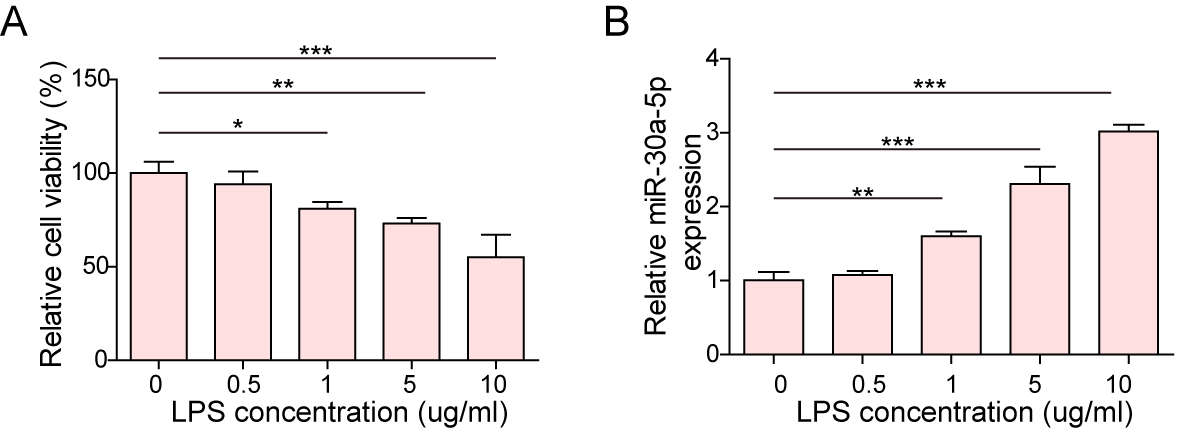

Supplement: Supplementary file 2 — Additional file 2: Supplementary Fig. 2. Effect of LPS on the viability and miR-30a-5p expression in GCs. GCs were exposed to LPS (0.5, 1, 5, 10 μg/mL) for 48 h. (A) The viability of GCs was assessed by CCK-8. (B) Expression of miR-30a-5p was detected by RT-qPCR. Results are presented as mean ± SD. n = 3. * p < 0.05, ** p < 0.01, *** p < 0.001. [file 13048_2024_1355_MOESM2_ESM.tif]
